# Evaluation of the Vitros® Anti-T. cruzi assay for its incorporation into the *Trypanosoma cruzi* infection diagnostic algorithm

**DOI:** 10.1016/j.heliyon.2024.e40673

**Published:** 2024-11-28

**Authors:** Miriam Campos-Ruiz, Jun Hao Wang-Wang, Belén Rivaya, Beatriz Rodriguez-Ponga, Noelia López, Victoria González, Pere-Joan Cardona, Gema Fernández-Rivas

**Affiliations:** a Microbiology Department, Clinical Laboratory North Metropolitan Area, Germans Trias I Pujol University Hospital, 08916 Badalona, Spain; b Department of Genetics and Microbiology, Autonomous University of Barcelona, 08916 Badalona, Spain; c Center for Epidemiological Studies on HIV/AIDS and STI of Catalonia (CEEISCAT), Generalitat de Catalunya, Ctra, Del Canyet, S/N, 08916 Badalona, Spain

**Keywords:** Chagas disease, *Trypanosoma cruzi*, Serological diagnosis, Native antigen

## Abstract

**Background and objective:**

Serological screening for Chagas disease (CD) in Latin American adults living in Europe is a cost-effective strategy for transmission prevention. The World Health Organization recommends two different serological tests including native and recombinant antigens for IgG detection. In Spain, most commercialized native tests require manual processing. We aimed to evaluate the sensitivity and specificity of the automated Vitros® Anti-T. cruzi native antigen-based test.

**Methods:**

A total of 556 serum samples were tested using two different tests: 1) our reference assay, a chemiluminescence immunoassay employing a recombinant multi-antigen protein (Liaison® XL Murex Chagas); 2) a chemiluminescence immunoassay with native antigen (Vitros® Anti-T. cruzi assay). Additionally, 180 samples were also processed by a manual indirect immunofluorescent assay (Chagas IFA). Sensibility, specificity and kappa index were calculated.

**Results:**

Vitros® showed a kappa index of 0.94 (IC 95 %: 0.86–1.03) compared to Liaison® XL with a sensitivity of 93.6 % and specificity of 99.5 %. Compared to IFA, Vitros® showed a kappa index of 0.61 (IC 95 %: 0.47–0.76), sensitivity of 97.5 % and specificity of 70.37 %. Discrepant results were obtained mainly in treated patients.

**Conclusions:**

The Vitros® Anti-T. cruzi assay showed potential for implementation as an automated serological screening test, enhancing the diagnostic process in high-throughput microbiology laboratories.

## 1 Introduction

*Trypanosoma cruzi* is a flagellate protozoan responsible of American Trypanosomiasis commonly known as Chagas disease (CD) [1]. The infection is presented by an acute phase, usually asymptomatic, followed by a chronic phase in untreated patients, in which approximately 10–30 % of cases may develop potentially life-threatening cardiac, gastrointestinal tract or nervous system disorders [2].

In Latin America (LA), where *T. cruzi* is mostly triatomine vector-borne [2], CD is considered one of the greatest public health problems of extreme seriousness. The World Health Organization (WHO) recognizes CD as one of the main neglected diseases [3]. Not only limited to LA, it is estimated that 75 million people are at risk of infection and 6 to 7 million people worldwide are infected with *T. cruzi* [4].

Due to population mobility and globalisation, the burden is growing in non-endemic countries [5] where transmission mainly occurs by blood transfusion and organ transplantation [6,7]. Over 4 million LA migrants are estimated to reside in Europe [8], and approximately 4.2 % of them are chronically infected with CD [7]. Spain has the highest seroprevalence of infected migrants in Europe (2.3 %–3.8 %) followed by Belgium, Italy and Switzerland with rates between 1.5 and 2.1 % [9].

In Spain, most patients present with the chronic and sometimes asymptomatic disease [10]. In this phase, parasitaemia is low and intermittent, making direct parasitological and PCR-based diagnostic methods unreliable [2]. Diagnosis of chronic infection depends on serological testing through detection of IgG antibodies against *T. cruzi*.

Traditional serological techniques used for CD diagnosis can be based on whole parasite antigens and purified extracts, (conventional tests) with the most common being indirect fluorescent assay (IFA), and enzyme-linked immunosorbent assay (ELISA); or on recombinant antigens and synthetic peptides (non-conventional tests) [11]. Serological diagnosis is limited due to the different immune response among patients and the potential cross-reactivity with other trypanosomatids or even parasites of the *Leishmania* genus coexisting in endemic areas when using native parasite antigens [12]. Moreover, the biological diversity and genetic polymorphism of *T. cruzi*, attributed to the existence of seven discrete typing units (DTUs) of the parasites may also involve false positive results [13]. Finally, tests using crude, native, or total antigens are considered to have more cross-reactions, resulting in lower specificity [11]. All together with the availability of multiple types of assays with variable sensitivity and specificity has contributed to the absence of a gold standard test for CD diagnosis. For these reasons, the WHO recommends using at least two tests with different antigens and/or principles in parallel to improve diagnostic accuracy [14]. When inconclusive or discordant results appear, a third technique or additional samples are required [14]. Since 2015, the WHO algorithm has been adopted in Catalonia and has been implemented in our Microbiology Department.

The increasing number of tests ordered for CD has forced high-throughput laboratories to adopt diagnostic algorithms with an automated recombinant antigen method with a tendency towards the use of a single test for CD diagnosis. Consequently, some fully automated chemiluminescence immunoassays have emerged in the recent years: Liaison® XL murex Chagas (DiaSorin, Italy) allows the qualitative detection of IgG anti *T. cruzi* and Chagas VirClia (Vircell, Spain) detects IgM and IgG antibodies against *T. cruzi*.

**Fig. 1:**
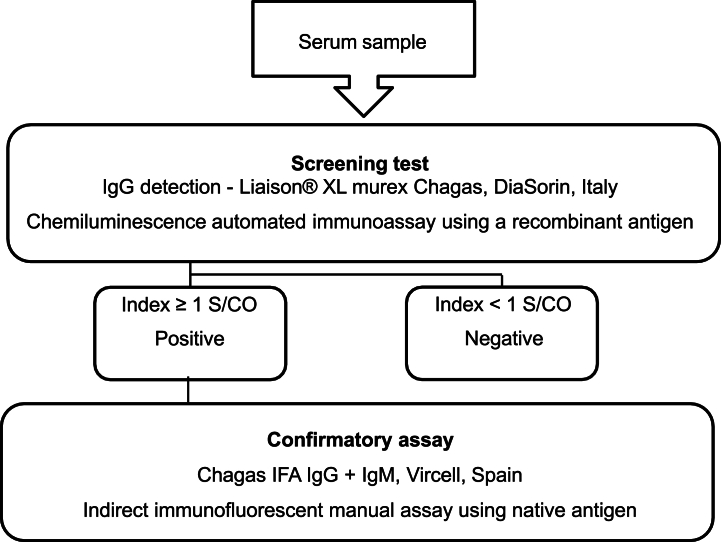
Diagnostic algorithm for Chagas disease performed at our setting. Serological diagnosis includes an automated screening test based on recombinant antigen and a confirmatory assay using a native antigen following the WHO recommendations.

Following the WHO recommendations, the diagnostic algorithm followed in our laboratory includes the recombinant antigen-based Liaison® XL murex Chagas as a screening method and a manual indirect immunofluorescence native assay Chagas IFA IgG + IgM (Vircell, Spain) as a confirmatory test (Fig. 1). The Chagas IFA IgG + IgM assay is time-consuming, laborious and requires trained technical staff for its correct interpretation.

Until a few years ago, most native tests marketed in Spain were not automated and manual processing was required. Nevertheless, in recent years some automated serological tests based on native antigen have become available in Europe. Vitros® Anti-T. cruzi automated test (Ortho-Clinical Diagnostics, USA) is an ELISA that uses MicroWells coated with a whole cell lysate containing *T. cruzi* native antigens as a solid phase. More recently, Chagas TESA VirClia® has been developed and uses excretion-secretion antigens of trypomastigotes for the first time in an automated assay [15]. Considering these automated tests based on native antigens, we aimed to evaluate the Vitros® Anti-T. cruzi detection test to consider its incorporation into the laboratory's daily routine as a screening test and secondly as a confirmatory test.

## 2 Material and methods

### 2.1 Study population and serum samples

A total of 556 serum samples from adults and one from an eight-month-old child admitted to the University Hospital Germans Trias i Pujol in Barcelona (Spain) were included in this study: 202 frozen samples for retrospective and 354 for prospective analysis, respectively. In both cohorts all Liaison® XL values and IFA results (if available) were included. Clinical data were obtained by reviewing the patients' medical records. Serum samples (preserved at −20 °C) were collected from 2016 to 2019. Those serums that are macroscopically turbid (milky) or hemolytic were excluded from the study.

### 2.2 Serological assays

All samples were tested in parallel by two different tests: 1) our reference assay based on recombinant antigen (Liaison® XL Murex Chagas, DiaSorin, Italy) (sensitivity: 99.1 %; specificity: 99.7 %); 2) a chemiluminescence immunoassay based on native antigen (Vitros® Anti-T. cruzi assay, Ortho-Clinical Diagnostics, USA) (sensitivity: 100 %; specificity: 99.9 %).

One hundred and eighty out of 556 samples were processed also by a manual indirect immunofluorescent assay (Chagas IFA IgG + IgM, Vircell, Spain) (sensitivity: 100 %; specificity: 98.6 %). According to our laboratory policy, patients who were already diagnosed with CD by two-step serology algorithm are followed-up with Liaison® XL murex Chagas to study the humoral kinetics. Hence, not all the samples were tested by IFA.

Liaison® XL Murex Chagas is a chemiluminescence immunoassay employing a recombinant multi-antigen protein representing 9 distinct antigenic regions designed to express different T. cruzi epitopes involved in the immune response. Vitros® Anti-T. cruzi ELISA uses whole parasite lysate antigens of *T. cruzi* *Tulaheun* strain. Chagas IFA IgG + IgM uses the *T. cruzi* epimastigotes fixed on a glass slide.

For Liaison® XL Murex Chagas and Vitros® Anti-T. cruzi the chemiluminescence reaction results were measured in relative luminescence units (RLU) and expressed as sample RLUs/cut-off value (S/CO). Samples with S/CO index ≥1 were considered positive and those with S/CO index <1 were considered negative (S/CO < 1) following the manufacturer's recommendations.

For IFA, a sample was considered positive when fluorescence was observed in the peripheral, cytoplasmic, and flagellar regions of the epimastigotes, and comparable fluorescence brightness as the positive control. Cases with lower or no brightness were considered negative, following the manufacturer's instructions. Each sample was reviewed by three different observers.

### 2.3 Data analysis

The following measures of diagnostic accuracy were calculated using the OpenEpi free software [16] (TP, true positive; TN true negative; FP, false positive; FN, false negative): sensitivity (calculated as TP/[TP + FN]; specificity (calculated as TN/[TN + FP] and kappa index, which is used to assess the inter-rater reliability or agreement. Prevalence of CD was calculated from the prospective cohort.

## 3 Results

The results obtained with Liaison® XL and Vitros® are summarised in Table 1. According to Liaison® XL, a total of 385 (69.24 %) serum samples were found to be negative for *T. cruzi* antibody detection and 171 (30.76 %) serum samples resulted positive. Using Vitros® assay, a total of 394 (70.86 %) samples resulted negative and 162 (29.14 %) were found to be positive.

**Table 1:** Results of *T. cruzi* serology obtained using Liaison® XL and Vitros® in 556 serum samples.

Serum samples (n = 556)		Vitros®	
		Negative	Positive
Liaison® XL	Negative	383	2
	Positive	11	160

Concordant negative for both Liaison® XL and Vitros® were obtained in 383 (68.9 %) samples whereas 160 (28.7 %) samples were positive in both assays with a mean index value of Liaison® XL and Vitros® of 11.19 S/CO and 6.73 S/CO respectively (data not shown). The concordant positive results obtained in the prospective cohort were 25 out of 353 resulting in a prevalence around 7 % of Chagas seropositivity in our area. Compared to our reference test Liaison® XL, Vitros® achieved a sensitivity of 93.6 %, specificity of 99.5 % and positive and negative predictive values of 98.8 % and 97.2 %, respectively. Vitros® showed a kappa index of 0.94 (IC 95 %: 0.86–1.03) with respect to Liaison® XL.

Discordant results between Liaison® XL and Vitros® were obtained in 13/556 (2.3 %) samples. The mean index value of Liaison® XL in the positive discordant patients was 2.74 S/CO. In those cases, the patient's clinical record was reviewed if available. Detailed information was shown in Table 2. Six out of 13 patients (46.2 %) were treated prior to the collection of the serum samples and therefore were previously diagnosed as CD; 5/13 (38.5 %) showed a negative confirmatory IFA assay; 1/13 (7.7 %) had a positive confirmatory IFA assay and 1/13 (7.7 %) was a leishmaniasis treated patient.

**Table 2:** Discrepant serological results obtained during the comparison of Liaison® XL and Vitros®.

Patient	Liaison		Vitros®		Plausible explanation
1	1.40	Positive	0.90	Negative	Treated (date unknown)
2	4	Positive	0.86	Negative	Treated in 2011
3	4.80	Positive	0.94	Negative	Treated in 2013
4	0.89	Negative	2.19	Positive	Treated in 2014
5	1.90	Positive	0.24	Negative	Treated in 2015
6	3.10	Positive	0.96	Negative	Treated in 2016
7	1.30	Positive	0.08	Negative	Negative confirmatory assay (IFA)
8	4.30	Positive	0.41	Negative	
9	3.30	Positive	0.20	Negative	Negative confirmatory assay (IFA). Similar result in a previous serum (3 months earlier)
10	2.70	Positive	0.04	Negative	
11	5.20	Positive	0.03	Negative	Negative confirmatory assay (IFA). Similar results in a previous serum (5 months earlier)
12	2.40	Positive	0.28	Negative	Positive confirmatory assay (IFA). No clinical data available
13	0.01	Negative	1.21	Positive	Potential cross-reaction in a leishmaniasis cured patient

The results obtained with Vitros® were also compared with those obtained with our routine confirmatory IFA assay (Table 3). According to IFA, 161 samples (89.44 %) contained *T. cruzi* antibodies and 19 (10.56 %) resulted negative. Using Vitros® assay, a total of 165 samples (91.67 %) were found to be positive and 15 samples (8.33 %) were found to be negative. Using Vitros® in parallel with IFA, concordant negative results were obtained in 11 (6.1 %) samples and positive concordant results were obtained in 157 (87.2 %) samples. Then, compared to our reference confirmatory IFA assay, Vitros® achieved a sensitivity of 97.5 %, specificity of 70.37 % and positive and negative predictive values of 95.3 % and 82.6 % respectively. Vitros® showed a kappa index of 0.61 (IC 95 %: 0.47–0.76) with respect to IFA. Twelve out of 180 serum samples (6.7 %) showed a discordant result between both methods (Table 4): 7/12 (58.3 %) patients were treated prior to the collection of the serum samples; 1/12 (8.3 %) was an 8-months healthy baby from a CD infected mother and in 4/12 (33.3 %) patients any information was available in their clinical record.

**Table 3:** Results of *T. cruzi* serology obtained using IFA and Vitros® in 180 serum samples.

Serum samples (n = 180)		Vitros®	
		Negative	Positive
IFA	Negative	11	8
	Positive	4	157

**Table 4:** Discrepant serological results obtained during the comparison of IFA and Vitros®.

Patient	Vircell IFA	Vitros®		Plausible explanation
1	Negative	1.43	Positive	8-months healthy baby from a CD infected mother
2	Negative	2.58	Positive	Treated in 2009
3	Negative	2.37	Positive	Treated in 2013
4	Positive	0.94	Negative	
5	Negative	3.69	Positive	Treated in 2015
6	Positive	0.24	Negative	
7	Negative	1.18	Positive	Treated in 2016
8	Positive	0.96	Negative	
9	Negative	1.52	Positive	No available information
10	Positive	0.28	Negative	
11	Negative	1.41	Positive	
12	Negative	1.62	Positive	

## 4 Discussion

Serological tests using crude antigens, such as IFA and ELISA, have historically been widely employed to detect *T. cruzi* antibodies. Until a few years ago, most native antigen-based tests accessible in Spain were not automated and manual processing was required. Then, it required trained staff, was time-consuming and may imply subjective interpretations of the results. To solve this problem, some automated serological tests based on native antigen have become available in Europe the past few years. In the present study, we aimed to evaluate the Vitros® Anti-T. cruzi assay which demonstrated a good analytical sensitivity and specificity compared to our routine assays. The Vitros® Anti-T. cruzi ELISA uses whole parasite lysate antigens of *T*. *cruzi Tulahuen* strain. The information provided by the manufacturer indicates a sensitivity of 100 % (determined by testing 106 positive specimens from individuals in Bolivia, Chile, Colombia and Nicaragua) and a specificity of 99.9 % [17]. The sensitivity in the present study was 93.6 % compared to recombinant antigen-based Liaison® XL and 97.5 % compared to IFA assay. The different sensitivity obtained in this work compared to those obtained in previous studies may probably be due to the studied patient's origin. We have analysed a large cohort of healthy and infected patients for a 3 years period, specifically 556 serum samples from adults who visited our hospital, including 202 retrospective samples and 354 prospective samples. Although most of the studied patients were probably from LA countries, we could not record this data. It has been described that the levels of antibody reactivity and clinical sensitivity were lowest in donors from Mexico and intermediate in those from Central America compared with those from South America, who displayed the highest sensitivity [18]. Vitros® Anti-T. cruzi assay showed an almost perfect concordance (kappa index 0.94) with our screening test, supporting its inclusion as an automated native antigen-based screening assay. Nevertheless, the agreement between Vitros® and IFA assay was lower but also substantial (kappa index 0.61).

During the comparison of Vitros® Anti-T. cruzi assay with Liaison® XL we found some discordant results. Six samples resulted positive with Liaison® XL and negative with Vitros®. Negative results in confirmatory IFA assay could be obtained in five out of six samples. Moreover, five patients who had been treated prior to serum collection showed positive results using Liaison® XL whereas Vitros® assay resulted negative. These results are consistent with recent studies that have observed chemiluminescence assays like Liaison® XL may detect anti-*T. cruzi* antibodies for a longer time than conventional ELISA or IFI, potentially limiting their utility for patient follow-up after treatment [19]. Another plausible explanation for serodiscordant results in treated patients is that they were in different stages of seroreversion, then discrepant results are more likely to occur [20].

It is well established the higher sensitivity of recombinant antigen-based tests in contrast with native antigen-based tests. The mean index value of Liaison® XL in these serodiscordant patients was 2.74 S/CO in contrast to the mean index value of positive concordant results (11.19 S/CO). These values probably indicate low levels of anti-*T. cruzi* IgG. According to previous works, serodiscordant patients with antibodies near to the detection limit only tested positive by the highest sensitive assays [21].

The manufacturer reported a 79 % cross-reactivity for antibodies to *Leishmania* spp., based on sera collected in India, where CD is not endemic [17]. Different studies have used native-antigen-based tests derived from *Tulahuen* strain to evaluate cross-reactivity using serum samples from patients in endemic areas for both Chagas and Leishmaniasis. The observed cross-reactivity ranges from 18 % to 55 % [17,22,23]. This underscores the importance of evaluating diagnostic tests in the specific regions where they will be applied [17,23] In our study, we identified one patient cured of leishmaniasis who showed negative results using Liaison® XL and low positivity using Vitros®. These results are according to previous studies which have described the fact that the use of recombinant antigen has helped to overcome the problem of cross-reactions described in conventional tests [17,23]. Additionally, ELISA based on the K39 protein from serum samples can be used, as this protein is a highly conserved, immunodominant repetitive epitope of kinesin across *Leishmania* spp. This test is known for its high specificity, so a negative result can reliably rule out the presence of antibodies against *Leishmania* spp.

When we compared both native antigen-based tests, we found that most of the discrepancies were found in treated patients, probably due to different stages of seroreversion as we previously mentioned. In one case we obtained negative IFA result and low positive Vitros® value in an 8-months healthy baby from a CD infected mother, but anti-*T. cruzi* antibodies were never detected in follow-up. It has been well established that infants born to mothers with CD should be monitored for at least their first 9 months of life. Initially, parasitological techniques are preferred due to higher effectiveness in infants during the acute phase. Serology becomes useful only after the ninth month, coinciding with maternal antibody disappearance. Early antibody detection is not indicative of infection, but monitoring reveals a gradual decline in maternal antibodies, particularly in uninfected cases [24]. Then, in our patient we were probably detecting maternal antibodies.

Our study has some limitations. One of the main limitations was the lack of the information in the clinical record of some patients and the impossibility of follow-up especially in serodiscordant patients. Additionally, in some frozen samples with discordant serological results, a third assay could not be performed as recommended by the WHO due to insufficient serum sample. Altogether made it difficult to determine exactly why some discordant results occurred. Finally, it was not possible to evaluate the cross-reactions with other protozoa antigens because only one serum sample from a single *Leishmania* spp.-infected patient could be obtained.

## 5 Conclusions

Based on our study, the Vitros® Anti-T. cruzi assay shows potential for being implemented as an automated serological screening test. This approach has the chance to streamline the diagnostic process in high-throughput microbiology laboratories by automating daily routines. Additionally, the use of native antigen-based Vitros® assay could aid in the follow-up of chronically infected Chagas patients, as a negative result may be indicative of potential cure.

## CRediT authorship contribution statement

**Miriam Campos-Ruiz:** Writing – original draft, Visualization, Validation, Supervision, Formal analysis. **Jun Hao Wang-Wang:** Writing – review & editing, Methodology, Investigation, Formal analysis, Data curation. **Belén Rivaya:** Writing – review & editing, Supervision, Resources, Methodology, Investigation, Formal analysis, Data curation, Conceptualization. **Beatriz Rodriguez-Ponga:** Writing – review & editing. **Noelia López:** Writing – review & editing, Methodology. **Victoria González:** Writing – review & editing, Methodology. **Pere-Joan Cardona:** Writing – review & editing. **Gema Fernández-Rivas:** Writing – review & editing, Supervision, Project administration, Methodology, Investigation, Conceptualization.

## Ethics statement

This study was approved by the Clinical Research Ethics Committee (CEIC) of the University Hospital Germans Trias i Pujol in Barcelona, Spain (PI-24-061).

In the present study, informed consent was waived since no additional samples were required for the evaluation, no clinical report was modified, and no patient data was used.

## Funding source

No funding source was involved.

## Declaration of competing interest

The authors declare that they have no known competing financial interests or personal relationships that could have appeared to influence the work reported in this paper.
